# Morphological Differentiation Towards Neuronal Phenotype of SH-SY5Y Neuroblastoma Cells by Estradiol, Retinoic Acid and Cholesterol

**DOI:** 10.1007/s11064-015-1743-6

**Published:** 2015-10-30

**Authors:** Heidi Teppola, Jertta-Riina Sarkanen, Tuula O. Jalonen, Marja-Leena Linne

**Affiliations:** Department of Signal Processing, Tampere University of Technology, P.O. Box 553, 33101 Tampere, Finland; Department of Cell Biology, School of Medicine, University of Tampere, Tampere, Finland; Department of Physiology and Neuroscience, St. George’s University School of Medicine, St. George’s, Grenada, West Indies

**Keywords:** Brain-derived neurotrophic factor, Cholesterol, Differentiation, Estradiol, Retinoic acid, SH-SY5Y

## Abstract

Human SH-SY5Y neuroblastoma cells maintain their potential for differentiation and regression in culture conditions. The induction of differentiation could serve as a strategy to inhibit cell proliferation and tumor growth. Previous studies have shown that differentiation of SH-SY5Y cells can be induced by all-*trans*-retinoic-acid (RA) and cholesterol (CHOL). However, signaling pathways that lead to terminal differentiation of SH-SY5Y cells are still largely unknown. The goal of this study was to examine in the RA and CHOL treated SH-SY5Y cells the additive impacts of estradiol (E_2_) and brain-derived neurotrophic factor (BDNF) on cell morphology, cell population growth, synaptic vesicle recycling and presence of neurofilaments. The above features indicate a higher level of neuronal differentiation. Our data show that treatment for 10 days in vitro (DIV) with RA alone or when combined with E_2_ (RE) or CHOL (RC), but not when combined with BDNF (RB), significantly (*p* < 0.01) inhibited the cell population growth. Synaptic vesicle recycling, induced by high-K^+^ depolarization, was significantly increased in all treatments where RA was included (RE, RC, RB, RCB), and when all agents were added together (RCBE). Specifically, our results show for the first time that E_2_ treatment can alone increase synaptic vesicle recycling in SH-SY5Y cells. This work contributes to the understanding of the ways to improve suppression of neuroblastoma cells’ population growth by inducing maturation and differentiation.

## Introduction

Neuroblastoma is the most common extra-cranial solid malignant tumor of sympathetic nervous system in infants and young children [1]. Regardless of its stage, until today, there is no cure or treatment, which could offer good prognosis for patients [2, 3]. Human SH-SY5Y cell line, used in this study, is a subclone of SK-N-SH cell line which was isolated from a bone marrow of a 4 year-old female patient [4]. SH-SY5Y cells maintain their potential for regression, which results in aggressive proliferation of these cells [5]. Novel therapeutic treatments inducing differentiation into neuronal cell types could help to improve the prognosis of children suffering from neuroblastoma [6]. The induction of differentiation could serve as a strategy to inhibit cell population growth and eventually stop the tumor growth, as well as induce healthy mature neurons in patients.

Previous studies have presented that differentiation of SH-SY5Y cells can be induced by dibutyryl cyclic AMP [5, 7], 12-*o*-tetradecanoyl-phorbol-13-acetate [8–13], all-*trans*-retinoic-acid (RA) [9, 14–16], brain-derived neurotrophic factor (BDNF) [17–19], vanadate [20], nerve growth factor [21, 22], cholesterol (CHOL) [23], vitamin D3, and neuregulin beta1 [24]. The signaling pathways that lead to terminal differentiation of SH-SY5Y cells, however, are still largely unknown.

The retinoic acid (RA) is a potent cell differentiating factor, which through its nuclear receptors affects a vast range of promoter sites in the neuronal and glial cells in every step of embryonic and postnatal life [25]. RA-induced differentiation has been shown to inhibit cell proliferation [9], change cellular sodium conductance [15], enhance the outgrowth of neurites [16], increase the acetylcholinesterase (AChE) activity [26, 27], and enhance the synaptic vesicle recycling [23]. However, clinical trials have demonstrated that treatment with RA alone, or in combination with interferon alpha, is not enough against recurrent neuroblastoma in children [28, 29]. Therefore, new alternative resources for more effective neuronal differentiation are needed.

Cholesterol (CHOL) is a necessary component in cell membranes and important for synaptic structure and function [30]. It is synthesized by neurons themselves for their survival and growth. The development of active synapses requires additional amount of cholesterol that is shown to be secreted by glial cells (specifically astrocytes) in the central nervous system [30–32] and by Schwann cells in the peripheral nervous system [33]. The glia-derived cholesterol has also been shown to be crucial for differentiation of dendrites, synaptogenesis, increase in synaptic protein expression (synapsin-1) and neuronal activity, and for transmitter release [30, 32, 34, 35]. In pure human SH-SY5Y cell cultures, the glia-derived cholesterol is non-existing, and addition of cholesterol is needed in order to achieve conditions resembling normal neuronal environment with surrounding glial cells [23].

The brain-derived neurotrophic factor (BDNF) has been shown to support the survival of neurons and stimulate the growth and differentiation of new neurons and synapses [36]. BDNF is a ligand for tropomyosin-related kinase B (TrkB) receptor, expression of which is lacking in naïve neuroblastoma cells. However, TrkB receptor expression and responsiveness to BDNF is induced by RA [37]. The activation of TrkB by BDNF has been suggested to enhance neuroblastoma cell survival and resistance to chemotherapy [38]. BDNF has also been shown to expose only a modest benefit for RA-induced arrest in a dormant state [6]. However, the sequential treatment of SH-SY5Y cells with RA and BDNF has been reported to induce differentiated, neurotrophic factor-dependent neuron-like cells [18] and sustained treatment has been reported to enhance neuronal differentiation of neural progenitor cells [39]. Moreover, RA-BDNF treatment induces significant increase in the expression of synaptic genes, brain miRNA, miRNA biogenesis machinery, and AChE activity, in comparison to sole RA treatment [19]. These studies stimulate the interest to further examine the potential therapeutic competence of BDNF for RA-induced SH-SY5Y cell differentiation and for treatment of neuroblastoma. Disruption of BDNF and its downstream signaling pathways have been observed in many neurodegenerative diseases such as Alzheimer’s, Parkinson’s and Huntington’s diseases [40–42], underlining the importance of BDNF. However, the results of the role of added exogenous BDNF for differentiation of RA treated SH-SY5Y cells are still controversial.

Estradiol (17-beta-estradiol, E_2_), a form of estrogen hormone, has both acute and long-term effects on a variety of neuronal cell types. Increase in the number of dendritic spines and number of excitatory synapses, which are the slow long-term effects of estradiol, were first detected [43–45]. The acute effects, which alter the intrinsic and synaptic physiology of neurons within minutes (reversible depolarization and increased input resistance with a latency of <1 min in 19.8 % of CA1 neurons tested) were detected later [46, 47]. Several studies have shown that estrogen enhances synaptogenesis and synaptic plasticity [48–55], which properties may be crucial for example in enhancing memory consolidation [43, 56]. Additionally, estrogen has been demonstrated to induce synaptic connectivity [52], enhance NMDA receptor expression and activity [57–66], and long-term potentiation (LTP) [58, 59, 64, 67]. Several earlier studies have addressed possible signaling mechanisms associated with estrogen-induced cellular functions. Estrogen activates these functions through the activation of estrogen receptors (ERs), ERα and ERβ, which serve as transcription factors modifying the activity of target genes [68, 69]. Estrogen has been shown to increase the key synaptic proteins, e.g. PSD-95, via either activation of ERα [70], ERβ [51], or both [71]. Estrogen is thought to use both nuclear ERs and plasma membrane ERs which are usually referred to as classical genomic and non-genomic pathways [69]. In classical genomic action, ERs are thought to translocate into nucleus in ligand-dependent manner and acting as a transcription factor of target genes after prolonged estrogen exposure [72]. In non-genomic action, estrogen has been shown to activate the membrane ERs, which rapidly stimulate the membrane-associated signaling molecules such as PI-3K and MAPK, resulting in quick increase in protein expression [73, 74]. SH-SY5Y cells have been shown to express both ERα and ERβ [75]. Estrogen has also been shown to stimulate the activity-regulated cytoskeleton associated protein (Arc) expression via the MAPK and PI-3K dependent pathway in SH-SY5Y cells [48]. Arc is known to be induced by neuronal activity and playing a key role in activity-dependent synaptic plasticity [76]. Its knockdown has been shown to lead to impairment of long-term memory [77, 78]. However, the specific role of E_2_ for inducing differentiation in human SH-SY5Y neuroblastoma cells is still unknown.

The main goal of the current study was to find a functional combination of substances for effective induction of differentiation of the SH-SY5Y cells. Based on our earlier studies, we used RA and CHOL as primary differentiation treatments [23]. We further investigated the ability of E_2_ and BDNF to support, and possible enhance, the RA and CHOL induced neuronal differentiation. We quantified the individual and additive impacts of BDNF and E_2_ on the RA and CHOL-induced neurite outgrowth, presence of neurofilament 68, synaptic vesicle recycling and arrest in the population growth rate of SH-SY5Y neuroblastoma cells in vitro.

## Methods

### Maintenance and Differentiation of Cell Cultures

The human SH-SY5Y neuroblastoma cell line (CRL-2266; American Type Culture Collection, Manassas, VA, USA) was cultured as previously described [23]. Briefly, the cells were plated at passage 29–30 with density of 5000 cells/cm^2^ on 48-well culture dishes. Cells were cultured and maintained in 5 % CO_2_ humidified incubator at 37 °C in 1:1 nutrient mixture F-12K Kaighn’s modification, and minimum essential medium supplemented with 10 % fetal bovine serum, 2 mM/L l-glutamine, 1 % antibiotic–antimycotic mixture and 1 % non-essential amino acids (all reagents from GIBCO, Invitrogen, Carlsbad, CA, USA, unless otherwise stated). Cell differentiation was induced with 10 µM/L all-*trans* retinoid-acid (RA), 1 nM/L 17-beta-estradiol (E_2_), 50 ng/mL brain-derived neurotrophic factor (BDNF), 10 µg/mL cholesterol (3β-hydroxy-5-cholestene, CHOL), or with combinations such as (i) 5 µM/L RA with 5 µg/mL CHOL (RC), (ii) 5 µM/L RA with 50 ng/mL BDNF (RB), (iii) 5 µM/L RA with 1 nM/L E_2_ (RE), (iv) 5 µM/L RA with 5 µg/mL CHOL, and 50 ng/mL BDNF (RCB), and (v) 5 µM/L RA with 5 µg/mL CHOL, 50 ng/mL BDNF, and 1 nM/L E_2_ (RCBE) for 10 DIV (all differentiation reagents from Sigma-Aldrich, St Louis, MO, USA, unless otherwise stated). Stock solutions of differentiation substances were diluted in 96 % ethanol; the final ethanol concentration never exceeded 0.1 % in cell culture. Control cells were treated with <0.1 % ethanol. All used substance concentrations were carefully evaluated according to already published literature. Suitable, least toxic concentrations, also used by other laboratories, were used to enable comparison of our results with others. All differentiation substances (except BDNF when used in combinations) were applied with medium exchange at 1, 3 and 7 DIV. BDNF was applied at 4 and 7 DIV when used together with RA (RB), RA and CHOL (RCB) or RA, CHOL and E_2_ (RCBE). The cell growth, condition, and morphology were observed with culture microscope (Olympus CK40) and images were taken at 10 DIV DP10 microscope digital camera system (Olympus, Tokyo, Japan).

### Neurofilament Staining

For detecting the level of differentiation in the neuroblastoma cell cultures, the cells were stained at 10 DIV with neuronal marker NF-68 for neurofilament light polypeptide (68 kDa, Sigma-Aldrich). Cells were first fixed for 20 min with 4 % paraformaldehyde (Sigma-Aldrich) in phosphate buffered saline solution (PBS), washed three times with PBS and permeabilized in 0.5 % Triton X-100 (J.T. Baker, Phillipsburg, NJ, USA) for 15 min. After washing with PBS, the non-specific antibody binding sites were blocked with 10 % bovine serum albumin (GIBCO) in PBS for 30 min to reduce the background. Cells were then incubated with the primary antibody mouse monoclonal anti-NF-68 1:200 for 1 h at room temperature (RT; +22 °C), rinsed three times with PBS, and then incubated with a secondary antibody FITC-conjugated goat anti-mouse IgG 1:100 (Sigma-Aldrich) for 30 min at RT. Fluorescence was visualized with Nikon Eclipse TS100 microscope equipped with Nikon DS Camera Control Unit DS L-1 and images were organized with Visio 2010 (Microsoft, WA, USA). The intensity of total neurofilament fluorescence (NF-68) and the intensity of total background fluorescence were measured from each fluorescence image with ImageJ software (National Institute of Mental Health, Bethesda, Maryland, USA) [79]. Corrected total neurofilament fluorescence (CTNF) was calculated from the gathered data in Excel 2010 (Microsoft, WA, USA) with the method used previously [80, 81], as follows: The fluorescence of the neurofilaments of interest was selected using the selection tool. Area of interest, integrated density, and mean gray value were calculated from selected areas with ImageJ software. A region next to the selected neurofilament was selected as a background value. The CTNF was calculated by using the following equation CTNF = integrated density − (area of selected neurofilaments × mean fluorescence of background readings).

### Quantification of Cell Population Growth

The substance-induced changes in the growth rate were quantified by counting the nuclei of 10 DIV cultured SH-SY5Y cells in each treatment group. Cell nuclei were stained with 10 µg/mL Hoechst 33258 (Sigma-Aldrich) for 5 min. Cultures were washed five times in PBS and mounted on cover slips. Fluorescence results were visualized with Nikon DS Camera Control Unit DS L-1. Images of each treatment group were analyzed with CellC analysis software [82], which corrects the image background for auto-fluorescence by fitting a two-dimensional quadratic polynomial to the image and subtracts the fitted polynomial surface from the original image. After this the algorithm separates the nuclei pixels from background pixels by global thresholding and produces a binarized image with white nuclei on a black background. It furthermore separates clustered nuclei from each other by marker-controlled watershed segmentation, which is based on nuclei intensity. Eventually the software removes artifacts, such as staining residues by discarding objects smaller than 1/10 of the mean size of all objects. Images were organized with Microsoft Visio 2010. The obtained nuclei counts and statistics (see section “Statistical Analysis”) were analyzed and plotted in MATLAB (version 2013b, The Mathworks Inc., MA, USA).

### Quantification of Neurite Length

The SH-SY5Y cells were cultured in CTRL, CHOL, E_2_, BDNF, RA, RE, RB, RC and RCBE conditions at 10 DIV. Neurites were traced from phase contrast images of each treatment group with NeuronGrowth plugin [83] of the ImageJ software (National Institute of Mental Health, Bethesda, Maryland, USA) [79]. The NeuronGrowth automatically counts the length of traced neurites in pixels and exports the data. The gathered data and statistics were analyzed and plotted in MATLAB (version 2013b, The Mathworks Inc., MA, USA).

### Synaptic Vesicle Recycling

The level of synaptic vesicle recycling was verified by measuring the number of fluorescence puncta in 10 DIV cultured SH-SY5Y cells. Cells were treated either with <0.1 % ethanol (CTRL), RA, CHOL, BDNF, E_2_, or with their combinations. Cultures were stained with AM1-43 styryl dye (Biotium, Hayward, CA, USA) for detecting synaptic exo/endocytosis in cells. AM1-43 is a fixable nerve terminal probe. It is not able to pass through the membranes, but instead, when cells are depolarized with high potassium (K^+^)-Tyrode solution, AM1-43 styryl dye attaches inside those vesicles, which are ongoing exocytosis. Staining was modified from method previously described [23, 84, 85]. In the current experiments, the cells were incubated for 1 min with 4 µmol/L AM1-43, according to manufacturer’s protocol, with the depolarizing Tyrode solution including 80 mmol/L K^+^ (80 mmol/L KCl, 29 mmol/L NaCl, 2 mmol/L MgCl_2_, 30 mmol/L glucose, 25 mmol/L HEPES, 2 mmol/L CaCl_2_). Cells were further washed several times with SCAS quencher solution (Biotium, Hayward, CA, USA) at RT to reduce background fluorescence. Cells were fixed for 20 min with 4 % paraformaldehyde (GIBCO), permeabilized in 0.01 % Triton X-100 (J.T. Baker) for 12 min and washed three times for 1 min in cold PBS. All reagents were from Sigma Aldrich unless otherwise stated. The fluorescence was visualized with Nikon Eclipse TS100 microscope equipped with Nikon DS Camera Control Unit DS L-1 and images were organized with Microsoft Visio 2010. Fluorescence images of each treatment group were analyzed with ImageJ analysis software [79] using the following procedural steps specifically designed to this study: background of the image was subtracted by setting a rolling ball radius to 50 pixels, after image was sharpened, and then the maxima of fluorescence puncta were found with noise tolerance of 20 and with the point selection style. This procedure was evaluated by visual inspection and it was found to be the best for finding the correct number of AM1-43 puncta from fluorescence images. The obtained counts of fluorescence puncta per image were divided by the obtained median nuclei number (see section “Quantification of Cell Population Growth”) in particular culture in 10 DIV treatments. These obtained counts of fluorescence puncta per median nuclei number and statistics (see section “Statistical Analysis”) were analyzed and plotted in MATLAB (version 2013b).

### Summary of the Level of Differentiation

Results are summarized in Table 1, which shows the level of differentiation induced by different treatments. The level of differentiation was assessed at least from three samples from two separate experiments by analyzing the following features; neurite length, presence of neurofilaments, inhibition in cell population growth rate and synaptic vesicle recycling. Neurite lengths were detected both visually and, by using automated methods to support the visual detection. Other features were defined according to the methods explained above (see sections “Neurofilament Staining, Quantification of Cell Population Growth and Synaptic Vesicle Recycling”).

**Table 1:** Summary of differentiation

Treatment^†^	Neurite length	Total neurofilament fluorescence	Inhibition of cell population growth	Synaptic vesicle recycling
CTRL	−	−	−	−
CHOL	++**	−	−	−
E_2_	+*	−	−	+*
BDNF	−	−	−	−
RA	+++**	+++**	+++**	++**
RE	+++**	++*	+++**	++**
RC	+++**	+++**	+++**	+++*
RB	+++**	+++**	++	++**
RCB	+++**	^‡^	^‡^	+++*
RCBE	+++**	+++**	^‡^	+++*

The criteria for categorizing the neurite length were as follows: − neurites similar to control, + short neurites without branching and significantly longer than in control [*p* < 0.05 (*)], ++ intermediate neurites without branching and significantly longer than in control [*p* < 0.01 (**)], +++ long neurites with branching and significantly longer than in control [*p* < 0.01 (**)] and in CHOL or E_2_ treatment conditions [*p* < 0.01 (**)]. The criteria for categorizing the total neurofilament fluorescence were as follows: − no neurofilament fluorescence, ++ significantly [*p* < 0.05 (*)] increased neurofilament fluorescence compared to control, +++ significantly [*p* < 0.01 (**)] increased neurofilament fluorescence compared to control. The criteria for categorizing the inhibition of cell population growth were as follows: − the number of cells has not changed after the treatment, ++ the number of cells decreased (not significantly), +++ the number of cells significantly [*p* < 0.01 (**)] decreased. The criteria for categorizing the amount of synaptic vesicle recycling were as follows: − no significant change in the counts of puncta in comparison to control, + significant [*p* < 0.05 (*)] increase in the counts of fluorescence puncta in comparison to control, ++ significant [*p* < 0.01 (**)] increase in the counts of fluorescence puncta in comparison to control, +++ significant [*p* < 0.05 (*)] increase in the counts of fluorescence puncta in comparison to RE and RB

^†^Control (CTRL, <0.1 % ethanol), cholesterol (CHOL; 10 µg/ml), 17-beta-estradiol (E_2_; 1 nM/L), brain derived neurotrophic factor (BDNF; 50 ng/mL), *all*-*trans* retinoic acid (RA; 10 µg/mL), RA with E_2_ (RE; RA 5 µg/mL, E_2_ 1 nM/L), RA with CHOL (RC; RA 5 µg/mL, CHOL 5 µg/mL), RA with BDNF (RB; RA 5 µg/mL, BDNF 50 ng/mL), RA with CHOL and BDNF (RCB; RA 5 µg/mL, CHOL 5 µg/mL, BDNF 50 ng/mL), RA with CHOL, BDNF and E_2_ (RCBE; RA 5 µg/mL, CHOL 5 µg/mL, BDNF 50 ng/mL, E_2_ 1 nM/L)

^‡^Conclusive data not available

### Statistical Analysis

Statistical analysis was performed using One-way ANOVA in MATLAB (version 2013b). Differences were considered to be significant when *p* < 0.01 or *p* < 0.05, different significances are indicated with ** or * in the figures, respectively.

## Results

### Morphology of Differentiated SH-SY5Y Cells

**Fig. 1:**
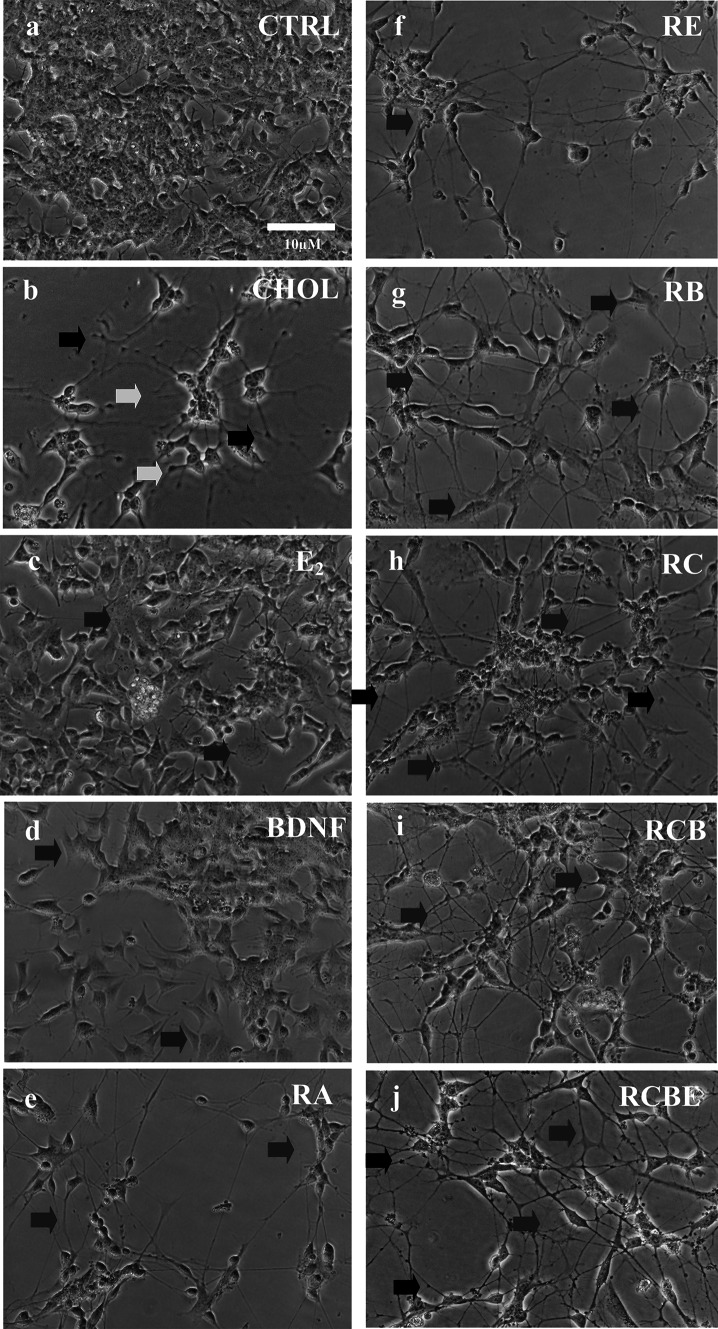
Morphology and network formation of SH-SY5Y neuroblastoma cells at 10 DIV. **a** SH-SY5Y cells were grown for 10 DIV in control conditions (CTRL) and with **b** cholesterol (CHOL), **c** estradiol (E_2_), **d** brain-derived neurotrophic factor (BDNF), **e** all-*trans* retinoic-acid (RA), or with their combinations **f**–**j** RE, RB, RC, RCB, RCBE, respectively. Data show that the CHOL treatment induced short neurites (*green arrows*) with many varicosities (*red arrows*) (**b**). E_2_ induced few very short neurites in comparison to CHOL induced neurites (**c**). BDNF treatment did not induce significant growth of neurites or network formation (**d**). RA treatment generated thin branching neurites and promoted network formation (*blue arrows*, **e**). Networks of cells with cell-to cell contacts (*blue arrows*) were always detected when treated with RA together with **f** E_2_ (RE), **g** BDNF (RB), **h** CHOL (RC), **i** CHOL and BDNF (RCB), and **j** CHOL, BDNF, and E_2_ (RCBE). Flat substrate-adherent (S-type) cells were detected especially when cells were treated with E_2_ or BDNF (*orange arrows*) (**c**, **d**) but also when cells were treated with RE (data not shown) or RB (*orange arrows*) (**g**). The RE treated cells (**f**) had thinner neurites than those treated with RCBE (**j**) (Color figure online)

Phase contrast images of SH-SY5Y cultures at 10 DIV were first visually analyzed for morphological assessment. In the visual analysis, control cells showed no particular neurite outgrowth (Fig. 1a), whereas CHOL-treated cells had a number of short neurites (green arrows in Fig. 1b; see also Table 1). Cells treated with E_2_ had very short neurites (Fig. 1c), which were both fewer and shorter than the CHOL-induced neurites. The morphology of BDNF treated SH-SY5Y was relatively polar and cells grew more spread in the culture dish (Fig. 1d). This differed from control cells, which grew in clusters (Fig. 1a). No significant outgrowth of neurites was observed after BDNF treatment. Treatment with RA alone (Fig. 1e) as well as with combinations such as RE, RB, RC, RCB or RCBE, induced branching of longer neurites and detectable network formation (Fig. 1f–j, respectively).

The data show other morphological differences between the treatments as well. The RE treated SH-SY5Y cells had networks of roundish cells with thin neurites without heavy branching (Fig. 1f). A small number of substrate-adherent (S-type) flat cells [86] were observed in cultures treated with RE (data not shown), RB (orange arrows in Fig. 1g) and RA (data not shown). The RB treatment induced networks that consisted of extended contacting neurites, as well as of cells in direct contact with each other without neurites. The neurites of the RB treated cells were thicker in comparison to RE induced neurites. The RC treatment induced cells with long, branching and connecting neurites (blue arrows in Fig. 1h). Other cholesterol treated cultures, such as RCB and RCBE, contained neurons with long branching neurites and network formation without S-type cells. More varicosities (red arrows in Fig. 1b) and small cell clusters (data not shown) were observed in CHOL treated cultures (CHOL, RC, RCB, RCBE) in comparison to the control, RE and RB treated groups, in which cells were more uniformly distributed (data not shown).

### Inhibition of Cell Population Growth

**Fig. 2:**
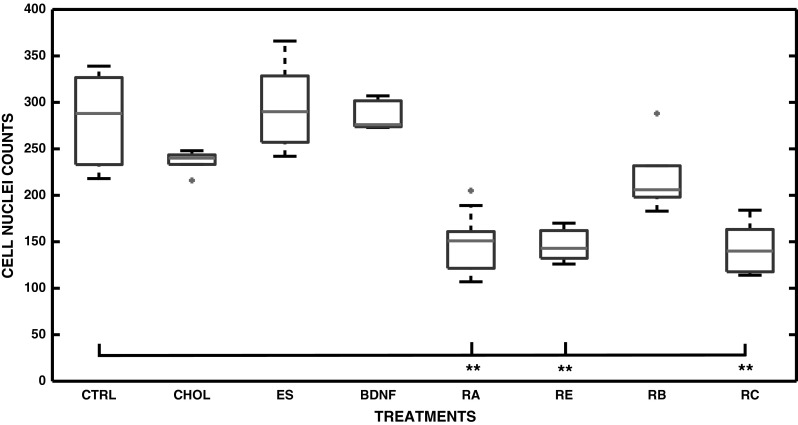
Inhibition of cell population growth. Cells were cultured in control conditions [CTRL (n = 7)], and with CHOL (n = 6), E_2_ (n = 6), BDNF (n = 6), RA (n = 6), RE (n = 6), RB (n = 6), and RC (n = 8) (n is the number of analyzed microscopy images) and the cell nuclei were counted at 10 DIV. In the *boxplot* representation the obtained median nuclei counts, 25th and 75th percentiles, extreme data points, and outliers of the data are shown with *red line*, *blue edges*, *black whiskers*, and *red asterisks*, respectively. Each differentiation agent is shown on the x-axis and the cell nuclei counts on the y-axis. The statistically significant differences (*p* < 0.01) are shown with *asterisks* (**). Significantly lower cell numbers were detected when cells were treated with RA, RE, or RC in comparison to CTRL data. Slight increase in cell numbers (nuclei counts) were observed when cells were treated with RB, in comparison to the cells treated solely with RA. The nuclei counts of RB treated cultures were not significantly lower in comparison to CTRL (Color figure online)

The ability of a substance to inhibit the population growth of human SH-SY5Y cells is one of the indicators of increased level of differentiation. Therefore, we counted the numbers of the Hoechst 33258 stained nuclei at 10 DIV in RA, CHOL, BDNF, E_2_, RE, RB, and RC treated cell cultures and compared the results to the number of nuclei in control conditions. The data demonstrated that CHOL, E_2_ or BDNF treatments on their own did not inhibit the cell population growth (Fig. 2), which, however, was seen when treated with RA, as well as with RA together with CHOL (RC) [*p* < 0.01(**)] when compared to controls. Moreover, significant (*p* < 0.01 (**) inhibition was also detected with RA together with E_2_ (RE) treatment, when compared to controls (Fig. 2). Interestingly, when cells were treated with RA and BDNF, no inhibition of growth was detected (Fig. 2).

### Neurite Lengths

**Fig. 3:**
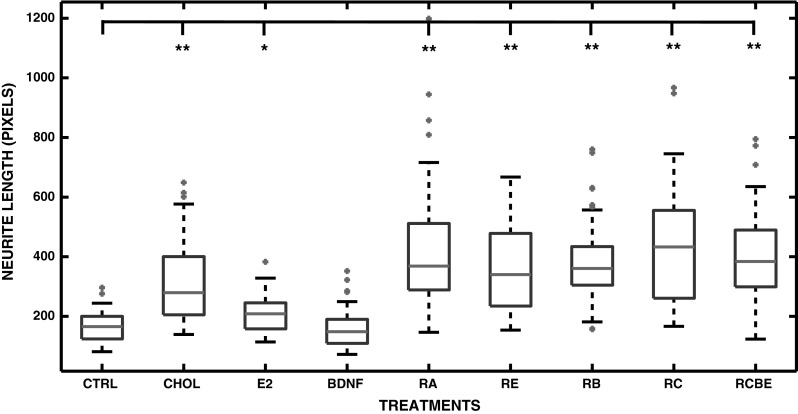
Neurite lengths in SH-SY5Y cells. Cells were cultured in CTRL conditions, and with CHOL, E_2_, BDNF, RA, or with their combinations; RE, RB, RC, RCBE. In the *boxplot* representation the obtained median of neurite lengths, the 25th and 75th percentiles, the extreme data points, and the outliers of the data are shown with *red line*, *blue edges*, *black whiskers* and *red asterisks*, respectively. Each treatment is shown on x-axis and the neurite lengths in pixels on y-axis. The statistically significant differences (*p* < 0.01) and (*p* < 0.05) are shown with *asterisks* (**) and (*), respectively. The neurite lengths were significantly longer when cells are treated with RA, CHOL, RE, RB, RC, and RCBE (*p* < 0.01). Interestingly, at 10 DIV, E_2_ induces only short neurites, but the increase in the neurite length is still significant in comparison to controls (*p* < 0.05). Moreover, RA induces significantly longer neurites when compared to CHOL or E_2_ induced neurite lengths (*p* < 0.01) (Color figure online)

The neurites of SH-SY5Y cells were traced from phase contrast images taken from each experiment at 10 DIV with NeuronGrowth (see section “Methods”), which provides supportive information of the lengths in addition to the visual inspection of the cell morphology. In addition to RA [*p* < 0.01 (**)], also with CHOL alone [*p* < 0.01 (**)] and E_2_ alone [*p* < 0.05 (*)] treatments, induced a significant increase in the length of neurites in comparison to control cells at 10 DIV (Fig. 3). Furthermore, the significant increase in the neurite length was seen in all combination treatments such as RE, RB, RC, RCBE [*p* < 0.01 (**)] relative to control conditions (Fig. 3). With the BDNF treatment alone no increase in the neurite length was detected.

### Presence of Neurofilaments in SH-SY5Y Neuroblastoma Cells

**Fig. 4:**
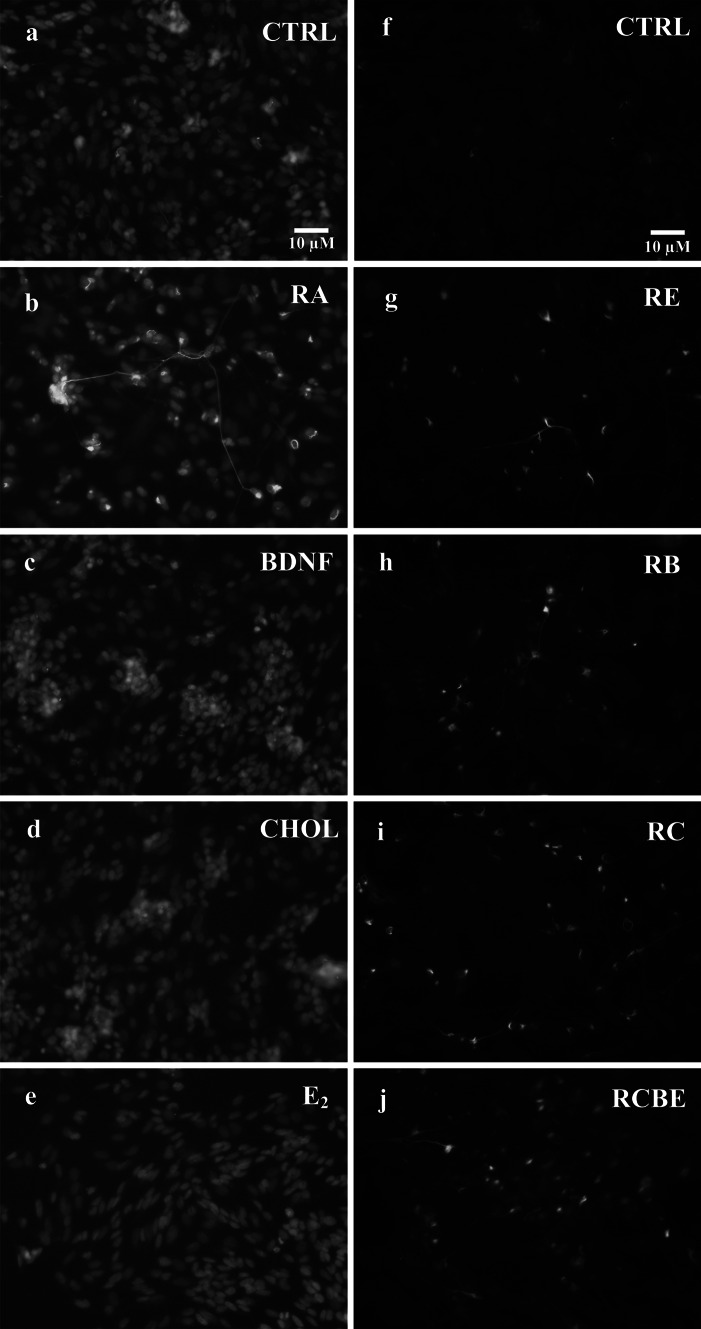
The presence of NF-68 neurofilaments in SH-SY5Y neuroblastoma cells. Neurofilaments were detected at 10 DIV by immunostaining of NF-68 (*green*) and cell nuclei with Hoechst 33258 (*blue*). **a**–**e** Combined double staining of neurofilaments and nuclei in the SH-SY5Y cells in control conditions (CTRL), and when treated with RA, BDNF, CHOL and E_2,_ show that RA induces NF-68, seen also in the long branching neurites. **a**, **f** No NF-68 is seen in CTRL. **g**–**j** RE, RB, RC and RCBE treatments show presence of neurofilaments similar to those with RA alone (Color figure online)

**Fig. 5:**
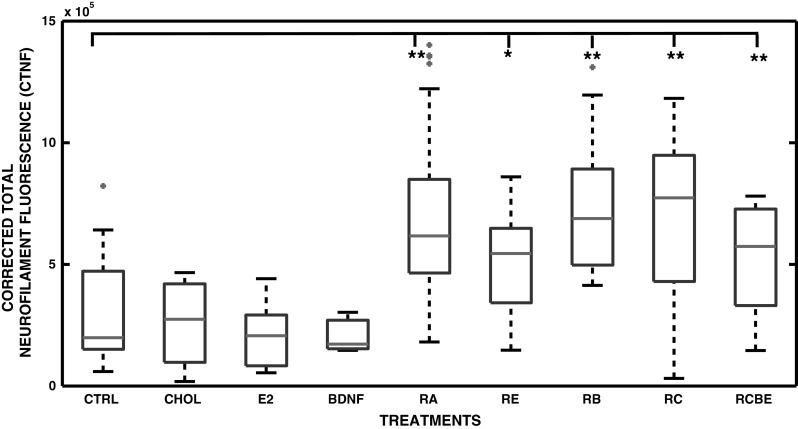
Corrected total NF-68 neurofilament fluorescence (CTNF). CTNF was analyzed from the control (CTRL) SH-SY5Y cells (n = 19), and cells treated with RA (n = 38), BDNF (n = 6), CHOL (n = 6) and E_2_ (n = 6) and RC (n = 9), RB (n = 14), RE (n = 13) and RCBE (n = 13) (n is the number of analyzed images). The obtained median of CTNF, the 25th and 75th percentiles, the extreme data points, and the outliers of the data are shown in the *boxplot* with *red line*, *blue edges*, *black whiskers* and *red asterisks*, respectively. Differentiation agents are shown on the x-axis and the CTNF on the y-axis. The statistical differences between the CTNF data and control values are shown with *asterisks* [*p* < 0.05(*), and *p* < 0.01(**)]. The automated analysis confirmed that the CTNF was significantly [*p* < 0.01(**)] increased in all other RA treated cultures (RA, RB, RC, RCB, and RCBE) in comparison to control cultures, except increased with less significance in the RE treated cultures [*p* < 0.05(*)]. This data show that NF-68 neurofilaments were induced by RA and further maintained by CHOL, BDNF and E_2_ (Color figure online)

The level of differentiation of the SH-SY5Y neuroblastoma cells at 10 DIV was further verified by imaging the NF-68 neurofilaments. The neurofilament fluorescence was defined by visual inspection and by measuring the intensity of CTNF, when cells were treated solely with RA, BDNF, CHOL, or E_2_ (Fig. 4b–e, respectively) or in combination with RA and E_2_ (RE), RA and BDNF (RB), RA and CHOL (RC), and RA, CHOL, BDNF, and E_2_ (RCBE) (Fig. 4g–j, respectively). Both visual and automated image analyses showed that the presence of the NF-68 neurofilaments was clearly induced by RA (Fig. 4b). No major increase in NF-68 fluorescence levels was observed visually or automatically, when cells were treated with BDNF, CHOL or E_2_ (Fig. 4c–e). However, the intensity of NF-68 fluorescence was significantly increased in all of the combination treatments relative to control cells, as shown in Fig. 4g–j, and in Fig. 5 for RA [*p* < 0.01 (**)], RE [*p* < 0.05(*)], RC [*p* < 0.01(**)], RB [*p* < 0.01(**)] and RCBE [*p* < 0.01(**)].

### Differentiation-Induced Synaptic Vesicle Recycling

**Fig. 6:**
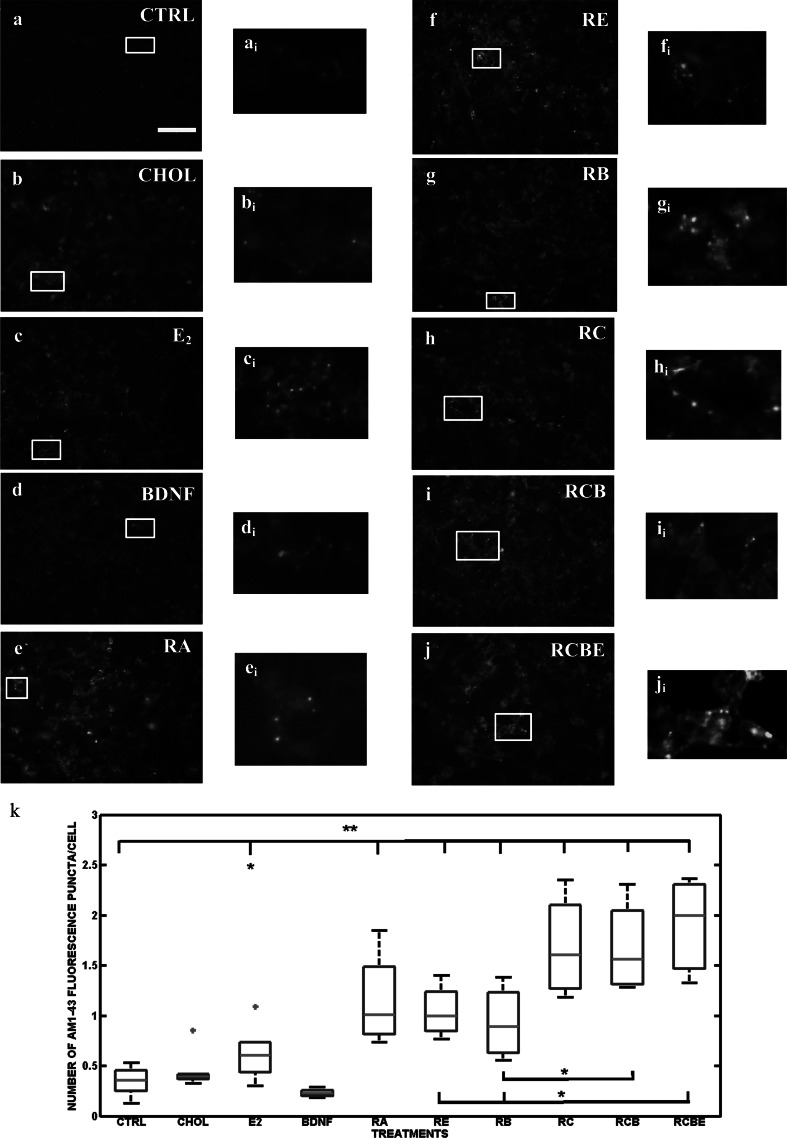
Synaptic vesicle recycling in SH-SY5Y neuroblastoma cells. **a**–**j** Synaptic vesicle recycling was measured by AM1-43 immunostaining in the presence of depolarizing high K^+^ containing Tyrode solution from control cells [CTRL (n = 12)] and cells treated with CHOL (n = 6), E_2_ (n = 6), BDNF (n = 6), RA (n = 6), or with their combinations such as RE (n = 6), RB (n = 6), RC (n = 6), RCB (n = 6), or RCBE, (n = 9) at 10 DIV (n is the number of analyzed images). **a**_**i**_–**j**_**i**_ Magnified examples of every treatment are shown. **k** Number of detected fluorescence puncta. In the *boxplot*, the obtained median number of fluorescence puncta, the 25th and 75th percentiles, the extreme data points, and the outliers of the data are shown with *red line*, *blue edges*, *black whiskers* and *red asterisks*, respectively. Differentiation agents are shown on the x-axis and the number of AM1-43 fluorescence puncta per obtained median nuclei number on the y-axis. The statistical differences between the data of interest and control values are shown as asterisks [*p* < 0.05(*), and *p* < 0.01(**)]. The number of fluorescence puncta did not significantly increase in comparison to CTRL group, when cells were treated solely with CHOL or BDNF (**b**, **d**, **k**). E_2_ induced significant increase [*p* < 0.05(*)] in the number of AM1-43 puncta relative to control group (**c**, **k**). The results show that RA alone, as well as RE, RB, RC, RCB, and RCBE significantly [*p* < 0.01(**)] increased the number of AM1-43 puncta relative to control group (**e**–**j**). Additionally, the synaptic vesicle recycling was significantly [*p* < 0.05(*)] increased when cholesterol was present (in RCB and RCBE treated cultures) when compared to RB or RE treated groups without CHOL (**k**). The highest median of number of fluorescence puncta was detected when neuroblastoma cells were treated with all substances simultaneously (RCBE) (Color figure online)

Our group has earlier shown that RA and RC treated human SH-SY5Y cells show intense Synaptophysin I (SypI) fluorescence in cell somata, along the neurites and at the sites of the cell-to-cell contacts. Furthermore, we have shown co-localization of SypI and AM1-43 at the end of the neurites at the cell-to-cell contacts of the RA and RC differentiated and high K^+^ depolarized human SH-SY5Y cells [23]. It has been also shown elsewhere that the SH-SY5Y cells are capable of depolarization with high K^+^ stimulation [87]. Therefore, high K^+^ stimulation was used for studying the stimulation-related synaptic vesicle recycling also in this study. The SH-SY5Y neuroblastoma cells were incubated with E_2_, CHOL, BDNF, or RA or with their combinations (RE, RB, RC, RCB and RCBE) and stained at 10 DIV with AM1-43, a fluorescent styryl dye (a nerve terminal probe) with the presence of depolarizing high K^+^-Tyrode solution. The number of fluorescent puncta, reflecting the recycling synaptic vesicles, was counted after depolarization (see Fig. 6 and section “Methods”). Treatment with CHOL or BDNF alone does not increase the number of fluorescence puncta in comparison to CTRL. Our data show for the first time, that the treatment with E_2_ alone [*p* < 0.05(*)], or RA together with E_2_ (RE), BDNF (RB), BDNF and CHOL (RBC) or BDNF, CHOL and E_2_ (RBCE), [*p* < 0.01(**)] significantly increases the number of fluorescence puncta when compared to controls. The results also confirm earlier results from our laboratory that RA alone, as well as together with CHOL, significantly [*p* < 0.01(**)] increases the number of AM1-43 fluorescence puncta in SH-SY5Y cells. This data also indicate a slight, however not significant, increase in fluorescence puncta in CHOL treated cultures, but not in BDNF treated cultures. In particular, we found a significant [*p* < 0.05(*)] increase in cells treated with RCB or RCBE relative to cultures treated without CHOL (such as RB or RE). These results demonstrate that in addition to RA and CHOL, also prolonged treatment with E_2_ alone stimulates the synaptic vesicle recycling in SH-SY5Y cells. Highest numbers of fluorescence puncta were detected when all agents were combined (RCBE).

## Discussion

Despite active research for improving the prognosis of high-risk neuroblastoma patients over the last decades, only a few patients become long-term survivors. Outcome of the stage 4 neuroblastoma remains poor, and the development of novel therapeutic approaches is needed [28, 88–91]. Therefore, therapies such as induced differentiation of cancer cells are important. Retinoic acid is one of the most prominent inducer of differentiation in clinical treatments [28, 90]. Therefore, in the current study, the level of differentiation of human SH-SY5Y cells towards neuronal type was followed by analyzing the additive impacts of estradiol (E_2_/E) and brain-derived neurotrophic factor (BDNF/B) on cell morphology, neurite length, presence of neurofilaments, cell population growth and synaptic vesicle recycling in non-treated controls, as well as retinoic acid (RA/R) and cholesterol (CHOL/C) treated cells at 10 days in vitro.

We here confirm our earlier data [23] that the most potent inducer for differentiation is retinoic acid, especially with co-treatment with CHOL. Of the four markers of differentiation (cell population growth, neurite length, total NF-68 fluorescence and synaptic vesicle recycling) growth is inhibited only when RA is present. Estradiol (E_2_), a predominant form of estrogen hormone, as well as CHOL, supports this inhibition. Growth rate of the cells treated with RA together with BDNF (RB) was higher than those, which were treated with RA alone. BDNF thus opposes the possibility of cells to undergo RA induced growth inhibition. Earlier results by other laboratories of the impact of BDNF, and in particular of the treatment together with RA, have given controversial results. While some studies show that the RB treatment differentiated the SH-SY5Y cells [18, 19, 37], other studies show that the activation of TrkB-BDNF pathway, which is also activated by RB treatment, can lead to increased cell survival, invasion, metastasis, angiogenesis and drug resistance [92–96]. Our results of population growth of RB treated cells are in agreement with the study by Cernaianu et al. [6], showing that the effect on proliferation depends on the used concentration of BDNF, causing either significant inhibition (with 10 ng/mL BDNF) or no inhibition (with 50 or 100 ng/mL) and with the study by Nishida et al. [97], where they show that SH-SY5Y-A cells (American Type Culture Collection; also used in this study) differentiated in the presence of RA whereas RA treated SH-SY5Y-E cells (European Collection of Cell Cultures) require additional BDNF treatment for full differentiation. This is due to the defects in several signaling pathways in SH-SY5Y-E cells. In conclusion, the controversial results in the level of differentiation in some of the studies might originate from the different subtypes of SH-SY5Y, as well as from the used BDNF concentrations. In the light of the current knowledge further examinations of the properties of BDNF are needed, before considering the use of BDNF as a therapeutic agent for clinical use.

Long neurites, typical of neuronal morphology, are found with treatment by retinoic acid, as well as by cholesterol. We here show, that E_2_ alone (at 1 nM concentration) is able to slightly increase the length of neurites, and when it is combined with RA (RE) or additionally with CHOL and BDNF (RCBE) neurites’ length is even more enhanced involving also branching and network formation. It has been earlier demonstrated by Takahashi et al. [98], that 10 nM E_2_ induces neurite outgrowth already after 2 DIV, which was not seen in this study. This difference in the presence of neurites may be explained by the 10 times difference in the E_2_ concentrations used. With treatment by BDNF alone, no neurite outgrowth at 10 DIV was detected. This result is understandable based on other reports that 3–5 days’ RA treatment is necessary to induce expression of TrkB receptors, which are crucial for BDNF induced neurite growth [18, 37]. Agholme et al. [24] have shown, that the RB and RCB treatments induced longer neurites than treatment with RA alone, which were not seen in our experiments. We additionally show, similarly to the results shown in previous studies, that RA alone [16, 23, 99] or when combined with CHOL (RC) [23], BDNF (RB) [100], or CHOL and BDNF (RCB) [24] induce considerable neurite outgrowth and neuronal networks.

Neurofilaments are major components of the neuronal cytoskeleton, providing structural support for the axons and regulating the axon’s diameter. We here show that treatment with RA alone enhances the total light neurofilament (NF-68) fluorescence. Earlier study from Messi et al. [101] is in agreement with our results by showing that RA reduces cell migration and invasiveness and up regulates NF-68 expression. In addition, it has been shown that medium size neurofilaments (NF-145-160) are induced by RA in SH-SY5Y cells [102]. We detected that RA combined to E_2_ enhanced the total NF-68 fluorescence, though less than the RC, RB, RCB or RCBE treatments. The enhancement of total NF-68 fluorescence in human SH-SY5Y cells by these treatments has not been previously reported.

Finally, we asked if RA, E_2_, CHOL and BDNF or their combinations increase the synaptic vesicle recycling in SH-SY5Y cells. Results from the current study are interesting, as in addition to RA, also E_2_, even when used alone, is able to significantly increase the number of detected vesicle recycling after depolarizing cells by high K^+^-solution. Hu et al. [103] have shown in cultured neonatal rat cortical primary cells that E_2_, produced and secreted by astrocytes, modulates synaptogenesis and synaptic function by increasing the synaptic vesicle recycling. In addition, they showed that added exogenic estradiol mimics this effect of astrocyte-conditioned medium on synaptic formation and transmission. Our results here are in agreement with this study. Chamniansawat and Chongthammakun [48] have shown that 48 h estrogen treatment significantly increases the expression of synapse related proteins, such as post-synaptic dense material 95 (PSD-95) and synaptophysin (SYP). As they did not see any estrogen induced immediate or rapid effects on the PSD-95 and SYP mRNA expression in SH-SY5Y cells, they concluded that the expressions of PSD-95 and SYP requires translocalization across ER, as well as long period of time to activate the target gene expression in SH-SY5Y cells. This is called classical genomic action of estrogen, where estrogen partially acts through PI-3K signaling to activate PSD-95 and SYP expression. Our data support the hypothesis that prolonged treatment of E_2_ enhances endo/exocytosis and thus promotes synaptic vesicle recycling in SH-SY5Y cells.

In the current study, synaptic vesicle recycling is significantly enhanced in all combination treatments, where cholesterol is present (RC, RCB and RCBE). Earlier it has been shown that the depolarized RC treated cells induced higher number of AM1-43 positive fluorescence puncta/cell in comparison to depolarized RA treated cells [23]. We now show for the first time that this same supra-additive effect can be observed in addition to depolarized RC treated cultures also in depolarized RCB and RCBE treated cultures. Sarkanen et al. [23] found that one aspect possibly explaining this effect of RA and CHOL is that the RA-induced fragmentation of Golgi apparatus is avoided by co-treatment with CHOL. In addition, it has been earlier shown that RA has antioxidant potential and, that RA isomers enhance the expression of genes linked with cholesterol efflux e.g. apoe, abca-1 and abcg-1 proteins in astrocytes [104]. We know also from other earlier studies that synaptogenesis is promoted by cholesterol [30], cholesterol influences multiple aspects of synaptic transmission [105], both presynaptically, by acting on neurotransmitter vesicle fusion [106–108] and postsynaptically, altering neurotransmitter receptor mobility in the membrane [109]. Moreover, it is known that retinoic acid has been shown to function in homeostatic plasticity as a signaling molecule that increases synaptic strength by a protein synthesis-dependent mechanism [110]. It is also known that homeostatic synaptic plasticity may manifest as altered presynaptic transmitter release and vesicle loading properties [111, 112]. All above mentioned studies support our current result of the supra-additive effect of cholesterol and retinoic acid on increasing the number of AM1-43 fluorescence puncta in SH-SY5Y cells when compared to cells treated without CHOL.

Earlier and our results indicate that the formation of new synapses is a complex process requiring the presence of multiple substances simultaneously. Cholesterol is especially important as a component of cellular membranes, regulating membrane structure, fluidity and permeability, and as a precursor for steroid hormones. The increased cholesterol efflux has been shown to impair the LTP at the hippocampal CA1 synapses [34]. This study, together with our results, indicates the importance of cholesterol in regulation of synaptic vesicle recycling, neurotransmission, and regeneration of synapses. Lately, research has also started to focus on the effects of estradiol (estrogen) on formation and activity of synapses. A recent study on adult male rat hippocampal slices shows that estradiol treatment enhances synaptic transmission and LTP via estradiol receptor beta (ERβ) stimulation. Estradiol activates the RhoA-GTPase signaling, which causes actin polymerization within dendritic spines. The study suggests that estradiol is able to increase the fast excitatory postsynaptic potentials and causes a reduction in the threshold for lasting synaptic changes. Results of the study further indicate that the estradiol (similarly to RA) activates the synaptic TrkB receptors needed for the effects of the BDNF [113].

It is important to review the capacity of neuroblastoma cells to differentiate into a neuronal cell type and link this differentiation to those factors, which are normally present in neuronal microenvironment. Amongst the various glial cells in central nervous system, astrocytes are known to release cholesterol and growth factors and thus promote different aspects of synapse development [30]. Neurons depend on import of cholesterol via lipoproteins [35] to effectively maintain development of new connections via dendrites, dendritic spines and synapses. The efficacy and stability of the pre-synaptic transmitter release largely depends on presence of cholesterol [32]. It is also known that neurons are able to convert glia-derived cholesterol to steroids, which then promotes synapse formation [114], and that astrocytes, in addition to cholesterol also produce and release estradiol, which enhances neurite growth [115] and increases synapse number and function [103, 116]. In this study, we investigated whether cholesterol or estradiol are able to increase synaptic vesicle recycling in human SH-SY5Y cells, and it was found that estradiol, even alone, is able to promote synaptic vesicle recycling in these cells. Cholesterol, in contrast, does that only when used together with retinoic acid. Our results indicate the importance of estradiol, cholesterol and retinoic acid in synaptic function.

The findings reported here have significance for understanding the effects of retinoic acid, cholesterol, estradiol and brain derived neurotrophic factor, either alone or in combinations in the process of SH-SY5Y neuroblastoma cell differentiation into neuronal cell type. More than one agent is clearly necessary to reach this goal in aim to benefit the differentiation induced therapies.

## Abbreviations

AChE: Acetylcholinesterase
Arc: Activity-regulated cytoskeleton associated protein
BDNF: Brain-derived neurotrophic factor
CHOL: Cholesterol
CO_2_: Carbon dioxide
CTNF: Corrected total neurofilament fluorescence
DIV: Days in vitro
E_2_: Estradiol
ER: Estrogen receptor
LTP: Long-term potentiation
NF-68: Neurofilament 68 kD
PBS: Phosphate-buffered saline
RA: All-*trans* retinoic acid
RB: All-*trans* retinoic acid with BDNF
RC: All-*trans* retinoic acid with CHOL
RCB: All-*trans* retinoic acid with CHOL and BDNF
RCBE: All-*trans* retinoic acid with CHOL, BDNF and E_2_
RE: All-*trans* retinoic acid with E_2_
RT: Room temperature
TrkB: Tropomyosin-related kinase B
